# Concomitant Psychiatric Symptoms in Neurological Outpatients

**DOI:** 10.3390/ijerph16050860

**Published:** 2019-03-09

**Authors:** Jarim Kim, Yerim Kim, Jong Seok Bae, Ju-Hun Lee, Hong-Ki Song

**Affiliations:** 1 School of Communication, Kookmin University, Seoul 02707, Korea; jrkim@kookmin.ac.kr; 2 Department of Neurology, Kangdong Sacred Heart Hospital, College of Medicine, Hallym University, Seoul 05355, Korea; jongseokbae@hallym.ac.kr (J.S.B.); leejuhun@hallym.ac.kr (J.-H.L.); hksong@hallym.ac.kr (H.-K.S.)

**Keywords:** anxiety, cognitive dysfunction, comorbidity, depression, outpatients, neurology

## Abstract

To estimate the prevalence of concomitant psychiatric disorders in neurological outpatients and to assess the value of simple screening questionnaires in the identification of psychiatric symptoms, we analyzed a total of 803 patients who visited neurology clinics with neurological symptoms over a six-month period. Using self-reported questionnaires, we assessed psychiatric symptoms, such as stress (Perceived Stress Scale, PSS), depression (Patient Health Question 9, PHQ9), and anxiety (Generalized Anxiety Disorder 7, GAD7). According to the disease subtypes, we analyzed the psychiatric scales based on gender and age group. The prevalence of psychiatric comorbidities was lowest in patients with cerebrovascular disease (CVD) and highest among patients with cognitive decline and epilepsy. The overall prevalence of psychiatric symptoms markedly decreased with age. This decline was statistically significant for all questionnaires (PSS ≥ 14, *p* for trend = 0.027; PQH9 ≥ 10, *p* for trend = 0.005; GAD7 ≥ 10, *p* for trend = 0.002) and was more pronounced in males. Considering the high incidence of undetected psychiatric comorbidities and their associated burden, proactive psychiatric management should be included in neurological care. Psychiatric questionnaires could also be an effective screening tool for identifying psychiatric symptoms accompanying neurological symptoms.

## 1. Introduction

Previous research has found that neurological patients show a high prevalence of psychiatric illness [1,2]. Clinical neurologists have found that a number of neurological diseases, including stroke, seizure, Parkinson’s disease, and multiple sclerosis, are commonly accompanied by psychiatric symptoms [3,4]. Although the relationship between neurological disease and psychiatric symptoms is unclear, the prevalence of depression in neurological patients is three times higher than in emergency room patients [5]. In the case of dizziness, psychiatric factors account for 20–50% of neurological symptoms [6], whereas stroke produces psychiatric symptoms due to specific brain lesions that regulate mood, emotion, and cognition [3]. According to previous studies, more than 50% of neurological patients have a mental illness that satisfies the criteria in Diagnostic and Statistical Manual of Mental Disorders, 4th edition (DSM-IV), with the most common symptoms being mood disorders, followed by cognitive and anxiety disorders [7]. Because these disorders can be treated with appropriate medication or counseling, neurologists should identify psychiatric symptoms in conjunction with neurological symptoms. However, psychiatric illness is often not fully considered when treating neurological outpatients [1,7]. For example, in a study conducted by the neurology department of a large hospital, 72% of psychiatric morbidity was neglected by neurologists [8].

Concomitant psychiatric symptoms are a major cause of disability and diminish the quality of life for patients [9]. They also cause a significant socio-economic burden [10]. To date, no study has evaluated the concomitant psychiatric symptoms present in subtypes of neurological diseases. Therefore, the aim of this study was to investigate the prevalence of concomitant psychiatric symptoms in neurological outpatients using screening questionnaires and to examine whether there was a difference in prevalence according to age and gender.

## 2. Materials and Methods

### 2.1. Study Population

This was a retrospective observational study. We analyzed a total of 803 consecutive patients aged 18 or older who visited neurology clinics with neurological symptoms over a six-month period. Patients aged < 18 years, those who did not consent to participate in this survey, and those who could not participate due to medical conditions (e.g., hand weakness, deafness, aphasia, or blindness) were excluded. The Institutional Review Board of Kangdong Sacred Heart Hospital approved the study protocol (IRB No. 2017-01-007-004), and written informed consent was obtained from all patients.

### 2.2. Clinical Information

In this study, neurological diseases were classified into the following subtypes: cerebrovascular disease, cord and peripheral neuropathy, headache and pain, dizziness, cognitive decline, movement disorder, epilepsy, and miscellaneous. Cerebrovascular diseases include stroke, transient ischemic attack, and intra- or extra-cranial arterial stenosis. The miscellaneous group included non-specific muscle twitching, general weakness, sleep problems, and demyelinating diseases. Because of the small sample size, demyelinating diseases, such as Guillain-Barre syndrome or multiple sclerosis, were not categorized as an independent disease group. We assessed conventional vascular risk factors (hypertension, diabetes, dyslipidemia, atrial fibrillation, and smoking), previous stroke history, coronary artery disease, thyroid disease, insomnia, and known psychiatric illnesses (anxiety and depression). Known psychiatric illnesses were defined based on previous diagnosis, the use of medication, or both.

Using self-reported questionnaires, we assessed stress (Perceived Stress Scale, PSS) [11], depression (Patient Health Question 9, PHQ9) [12,13], and anxiety (Generalized Anxiety Disorder 7, GAD7) [14]. Based on previous studies, we set cut-off values for moderate to severe levels as follows: PSS ≥ 14 for perceived stress, PHQ9 ≥ 10 for depression, and GAD7 ≥ 10 for anxiety [11,13,14]. The PSS comprises 10 items that include both negative (Q1, 2, 3, 6, 9, and 10) and positive (Q4, 5, 7, and 8) items rated from “0” (never) to “4” (very often). In 100 participants from Athens and Larissa, the PSS-14 had a sensitivity of 78% and a specificity of 61% [15]. The PHQ9 is a self-reported survey that scores each of the 9 criteria as “0” (not at all) to “3” (nearly every day). According to a sample of 580 patients, PHQ9 ≥ 10 had a sensitivity of 88% and a specificity of 88% for major depression [12]. The GAD7 is a seven-item, self-reported questionnaire used to assess a subject’s mental status during the previous two weeks. Scores of “0” (no at all), “1” (several days), “2” (more than half the days), and “3” (nearly every day) are given. Using a cut-off score of 7–10, a previous study reported that GAD7 had a sensitivity of 83% and specificity of 84% [16]. These scales are commonly used screening methods and their validity has been established for Korean subjects. For example, of the total of 402 patients with chronic disease, the overall Cronbach’s α for the PSS was 0.75 (*n* = 402) [11], and among participants with migraine, the Cronbach’s α for the PHQ9 and GAD7 was 0.894 (*n* = 132) and 0.915 (*n* = 146), respectively [13,14]. We used these scales because of their simplicity, validity, and utility with Korean patients [11,13,14].

To determine age-dependent differences, we divided the patients into 30-year groups (i.e., < 30 years old, 30–59 years old, and ≥ 60 years old) and then compared psychiatric scores according to disease subtype, gender, and age group.

### 2.3. Statistical Analyses

Analysis of the demographic data was conducted using chi-squared tests or Student’s *t*-tests. For the chi-squared tests for trend, linear-by-linear association was used. The statistical significance of the differences between age groups was tested using one-way analysis of variance (ANOVA) and Scheffe’s test for multiple comparisons. Because some variables do not meet the chi-square assumption that 80% of the cells have an expected count over 5, we conducted Fisher’s exact test for those variables. A probability of ≤ 0.05 was considered significant. The analyses were performed using SPSS 21.0 (IBM, Armonk, NY, USA).

## 3. Results

### 3.1. Analysis I: Psychiatric Illness by Neurological Disease Subtype

**Figure 1:**
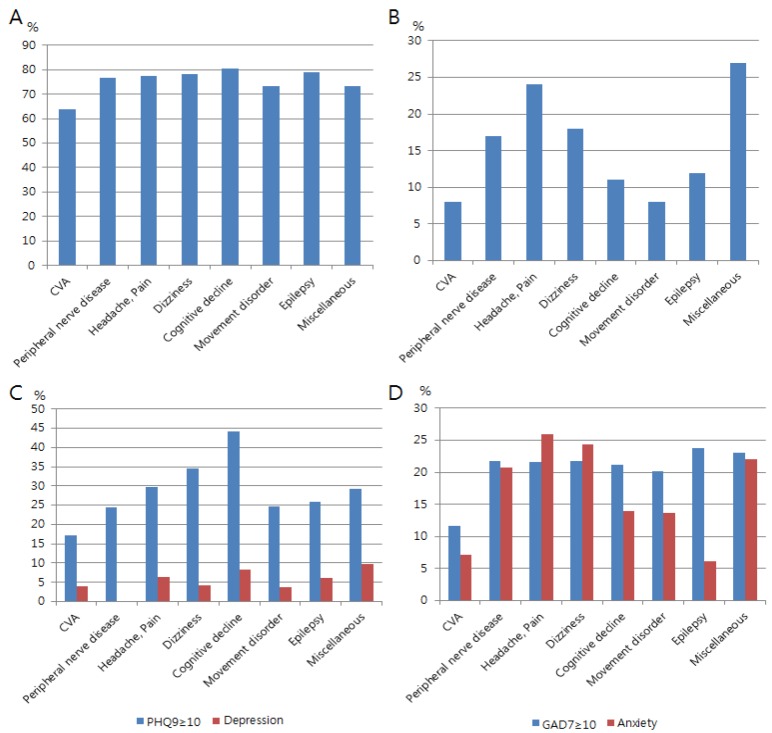
Psychiatric symptoms according to neurological disease subtypes: (**A**) moderate to severe perceived stress scale (PSS ≥ 14); (**B**) frequency of insomnia; (**C**) moderate to severe depression (PHQ9 ≥ 10, blue bars) and diagnosed depression (red bars); (**D**) moderate to severe anxiety (GAD7 ≥ 10, blue bars) and diagnosed anxiety (red bars).

In Table 1, patients with headaches or pain were female-predominant, while patients with CVD and cognitive decline were generally older. Moderate to severe perceived stress (PSS ≥ 14) was frequently reported in subjects with cognitive decline (81%), epilepsy (79%), headache and pain (78%), and dizziness (78%), while it was the least common in patients with CVD (64%; Figure 1A,B). Moderate to severe depression (PHQ9 ≥ 10) was most commonly reported in patients with cognitive decline (44%), dizziness (34%), and headaches and pain (30%; Figure 1C). Moderate to severe anxiety (GAD7 ≥ 10) was reported in patients with epilepsy (24%), dizziness (22%), headache and pain (22%), and cognitive decline (21%; Figure 1D).

It was found that anxiety and depression were relatively less frequently diagnosed when compared to the self-reported scores on the psychiatric scales (Table 1 and Figure 1A–D). This discrepancy between the self-reported scales and diagnosis was more noticeable for patients with depression (and PHQ9) than for anxiety (and GAD7; Figure 1C,D). In particular, for those patients with epilepsy, the prevalence of self-reported anxiety (GAD7≥ 10) was significantly higher than diagnosed anxiety (24% versus 6%; Table 1 and Figure 1D).

### 3.2. Analysis II: Psychiatric Illness Prevalence by Gender and Age Group

**Figure 2:**
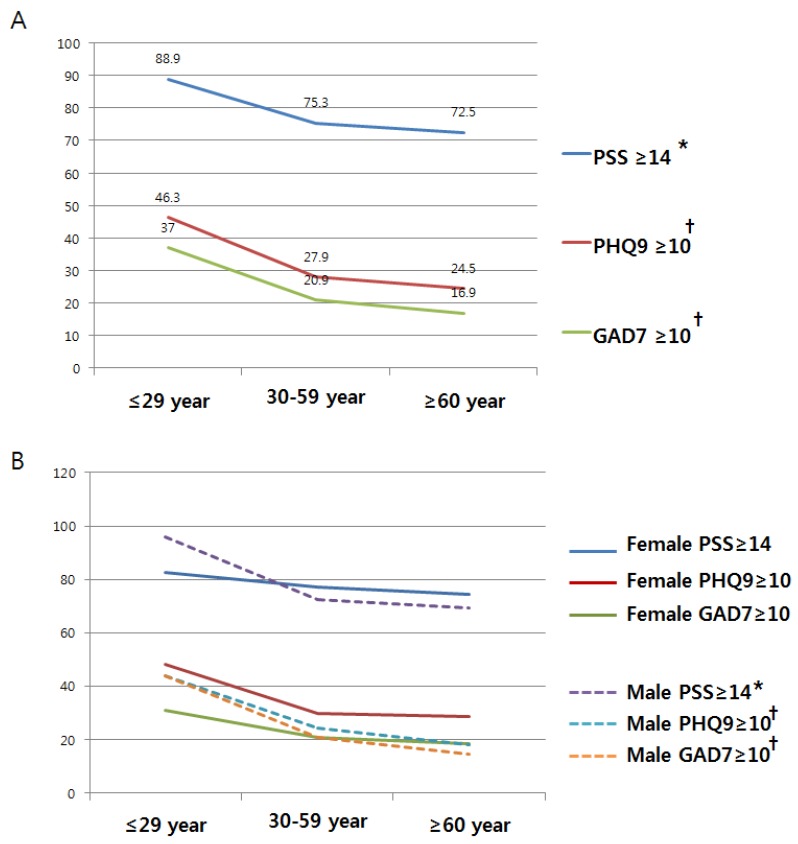
Psychiatric illness prevalence rates for the three self-reported psychiatric scales: (**A**) prevalence rates by age group; (**B**) prevalence rates by gender and age group. Note: * *p* < 0.05, ^†^ *p* < 0.01.

The overall prevalence of psychiatric illness decreased markedly with increasing age (Table 2). As shown in Figure 2A, these declines were statistically significant for all three psychiatric scales (PSS ≥ 14, *p* for trend = 0.027; PQH9 ≥ 10, *p* for trend = 0.005; GAD7 ≥ 10, *p* for trend = 0.002). This pattern was more pronounced for males (Figure 2B and Table 3).

## 4. Discussion

For neurological outpatients, psychiatric illnesses are common comorbidities. Recently, there has been a number of reports about the psychiatric symptoms associated with stroke and the need for appropriate treatment [17,18]. In our study, self-reported perceived stress, depression, and anxiety were lowest in patients with CVD and highest in patients with cognitive decline or epilepsy. The self-reported psychiatric scores were also significantly lower with an increase in age, and this was more pronounced for men than for women.

One point of note is that the prevalence of psychiatric symptoms was lowest in patients with CVD. The most common psychiatric issues after a stroke are depression, mania, anxiety, labile emotions [19] and suicidality [20]. According to a previous report, the prevalence of post-stroke depression ranges from 5 to 63%, peaking at 3 to 6 months after the onset of symptoms [21]. In addition, anxiety disorder was found in 30% of patients two days after an acute stroke and in 25% of patients 15 days after symptom onset [22]. This means that after a stroke, psychiatric symptoms change over time. Because the disease-onset time differs in the neurological outpatient’s department, the effect of time on psychiatric comorbidities may differ for this group of patients. In addition, the CVD group includes patients with asymptomatic intra- or extra-cranial arterial stenosis. This heterogeneity among patients may have lowered the prevalence of self-reported psychiatric disorders.

Another interesting point is that the prevalence of psychiatric symptoms detected using self-reported questionnaires in the present study significantly decreased with age. In addition, when comparing the subjects by gender, this trend was found to be stronger for males. This is somewhat surprising because it could be assumed that psychiatric illnesses would be more frequent among the elderly due to the natural changes to the body associated with aging increasing the likelihood of psychiatric illness. It is not known whether the trend observed in our study is specific to neurological patients or found in the general population. Similar to the results of our study, according to a nine-year retrospective observational study, medically unexplained physical symptoms were most frequently observed in the 15–45-year age group [2]. In addition, in a study of 198 neurological patients, overall psychiatric morbidity significantly reduced with increasing age [23], and in 294 patients with different diseases, including hematological, endocrine, eye, ear, skin, digestive system, musculoskeletal, and genitourinary disease, infections, malignancies, and injury, similar patterns were observed [24]. Furthermore, from the 2007 National Survey of Mental Health and Wellbeing of adults, it was found that the prevalence of mental disorders was highest in the 25–34-year age group and decreased with increasing age to 6% in the 75–85-year age group [25]. One hypothesis for our findings is that young adults may experience more severe occupational or economic stress in Korea compared to more stable older groups. For example, the employment rate of young adults is currently very low due to the economic recession.

Another possible explanation of why younger people scored higher in these scales is that psychiatric presentation reflects how well subjects manage it. Because PSS, PHQ9, and GAD7 are self-reported scales, if young patients are vulnerable to psychiatric pressure, it would be reflected in our results. Indeed, South Korea has had the highest suicide rate over the last 10 years among Organization for Economic Co-operation and Development (OECD) countries [26]. Based on the Statistics Act and Act on the Registration of Family Relationships, the most common cause of death was cancer in the 1–9-year age group and those aged 40 and over. However, in the 20–39-age group, suicide was the leading cause of death in 2014 [27]. Although the association between age and psychiatric symptoms is unclear, we suggest that psychiatric vulnerability might have affected the results.

Finally, there was a larger discrepancy between PHQ9 scores and diagnosed depression than GAD7 scores and diagnosed anxiety. This may be because clinicians can detect a patient’s anxiety more easily than depression because the latter has more negative symptoms, such as a loss of interest, a loss of energy, and the diminished ability to think [28]. If physicians do not pay attention to these negative symptoms, depression may be under-diagnosed. However, because anxiety disorders are often linked to depression, it is important that both conditions are managed simultaneously [29]. It also remains to be determined why subjects with epilepsy presented with a high prevalence of self-reported anxiety. In our study, the prevalence of self-reported moderate to severe anxiety was consistent with the findings of a previous study, which reported that the prevalence of anxiety related to epilepsy was 10–25%. Though the etiology has not yet been elucidated, physicians have speculated that it is related to the fear of an unpredictable loss of control due to their epilepsy [30].

To date, no study has investigated psychiatric illness in neurological outpatients based on disease subtypes using validated questionnaires. Because validated assessments can increase the reliability of a study [9], researchers should use these tools appropriately. This study, by investigating psychiatric illness by disease type, age, and gender, provides novel information for the treatment of patients in clinics. Despite these strengths, this study has some limitations. For example, the self-reported surveys may have led to response biases. Respondents may have felt time pressure or tired, and they may have attempted to achieve consistency rather than considering specific questions [31]. In addition, because this was a retrospective observational study, we could not compare the characteristics of the participating and non-participating patients. Finally, known psychiatric illness was not defined based on the 10th revision of the International Statistical Classification of Diseases and Related Health Problems (ICD-10), and we do not know how those illnesses were initially diagnosed. However, we did assess the psychiatric medication history of all patients.

## 5. Conclusions

Comprehensive management of concomitant psychiatric illness is important for more effective neurological care. However, psychiatric interviews with patients are often difficult to carry out in a busy clinical setting due to time limitations. The screening questionnaires used in this study had a high sensitivity and specificity despite their simplicity. Therefore, considering the high prevalence of undetected psychiatric symptoms associated with neurological disorders, these simple psychiatric scales are expected to benefit patients and healthcare providers by identifying the presence of psychiatric comorbidity.

## Figures and Tables

**Table 1:** Differences in frequency and descriptive statistics across disease subtypes ^1^.

Disease Subtypes	CVA (*n* = 125)	Peripheral Neuropathy (*n* = 82)	Headache, Pain (*n* = 174)	Dizziness (*n* = 148)	Cognitive Decline (*n* = 36)	Movement Disorder (*n* = 131)	Epilepsy (*n* = 66)	Miscellaneous (*n* = 41)	*p*-Value
Female	53 (42)	43 (52)	129 (74)	96 (65)	24 (67)	76 (58)	37 (56)	25 (61)	<0.001
Age (mean ± SD)	66 ± 12	58 ± 14	52 ± 14	55 ± 16	73 ± 9	64 ± 15	44 ± 17	54 ± 15	<0.001 *
T ^†^	a, b	b, c	c, d	c	a	b	d	c	
PSS ≥ 14	80 (64)	63 (77)	135 (78)	116 (78)	29 (81)	96 (73)	52 (79)	30 (73)	0.136
PHQ9 ≥10	21 (17)	19 (24)	51 (30)	50 (34)	15 (44)	31 (25)	17 (26)	12 (29)	0.021
GAD7 ≥10	14 (12)	17 (22)	37 (22)	31 (22)	7 (21)	25 (20)	15 (24)	9 (23)	0.456
Insomnia	10 (8)	14 (17)	41 (24)	26 (18)	4 (11)	10 (8)	8 (12)	11 (27)	<0.001
Known anxiety	9 (7)	17 (21)	45 (26)	36 (24)	5 (14)	18 (14)	4 (6)	9 (22)	<0.001
Known depression	5 (4)	0 (0)	11 (6)	6 (4)	3 (8)	5 (4)	4 (6)	4 (10)	0.125 ^‡^
Hypertension	73 (58)	37 (45)	36 (21)	54 (36)	18 (50)	54 (41)	9 (14)	11 (27)	<0.001
Diabetes	26 (21)	22 (27)	14 (8)	19 (13)	12 (33)	24 (18)	6 (9)	8 (20)	<0.001
Dyslipidemia	66 (53)	17 (21)	20 (11)	21 (14)	10 (28)	16 (12)	5 (8)	10 (24)	<0.001
Atrial fibrillation	8 (6)	0 (0)	1 (1)	2 (1)	0 (0)	2 (2)	0 (0)	1 (2)	0.020 ^‡^
Smoking	22 (18)	16 (20)	23 (13)	24 (16)	3 (8)	17 (13)	12 (18)	12 (29)	0.187
Previous stroke	104 (83)	1 (1)	3 (2)	7 (5)	4 (11)	10 (8)	8 (12)	0 (0)	<0.001
Coronary artery disease	12 (10)	6 (7)	5 (3)	8 (5)	2 (6)	10 (8)	0 (0)	4 (10)	0.042 ^‡^
Thyroid disease	3 (2)	4 (5)	3 (2)	7 (5)	3 (8)	11 (8)	4 (6)	0 (0)	0.058 ^‡^

^1^ Abbreviations: SD, standard deviation; PSS, perceived stress scale; PHQ9, patient health question 9; GAD7, generalized anxiety disorder 7; CVA, cerebrovascular attack; * Statistical significance was tested using one-way analysis of variance between groups; T, Test for post-hoc analysis; ^†^ The same letters indicate a non-significant difference between groups based on Scheffe’s multiple comparison test; ^‡^ Statistical significance was tested using Fisher’s exact test among groups; Percentages are presented in parentheses.

**Table 2:** Association between age group and psychiatric illness ^1^.

Age	≤29 Years	30–59 Years	≥60 Years	*p*-Value
PSS ≥ 14	48 (88.9)	274 (75.3)	279 (72.5)	0.033
PHQ9 ≥ 10	25 (46.3)	100 (27.9)	91 (24.5)	0.003
Known depression	3 (5.6)	19 (5.2)	16 (4.2)	0.753
GAD7 ≥ 10	20 (37.0)	74 (20.9)	61 (16.9)	0.002
Known anxiety	6 (11.1)	72 (19.8)	65 (16.9)	0.241
Insomnia	9 (16.7)	63 (17.3)	52 (13.5)	0.344

^1^ Abbreviations. PSS, Perceived Stress Scale; PHQ9, Patient Health Question 9; GAD7, Generalized Anxiety Disorder 7.

**Table 3:** Association between gender and psychiatric illness^1^.

Gender	Females	Males	*p*-Value
PSS ≥ 14	368 (76.2)	233 (72.8)	0.28
PHQ9 ≥ 10	144 (30.5)	72 (23.1)	0.023
Known depression	31 (6.4)	7 (2.2)	0.006
GAD7 ≥ 10	94 (20.3)	61 (19.8)	0.854
Known anxiety	99 (20.5)	44 (13.8)	0.014

^1^ Abbreviations: PSS, Perceived Stress Scale; PHQ9, Patient Health Question 9; GAD7, Generalized Anxiety Disorder 7.
